# ESGAR consensus statement on the imaging of fistula-in-ano and other causes of anal sepsis

**DOI:** 10.1007/s00330-020-06826-5

**Published:** 2020-04-19

**Authors:** S. Halligan, D. Tolan, M. M. Amitai, C. Hoeffel, S. H. Kim, F. Maccioni, M. M. Morrin, K. J. Mortele, S. R. Rafaelsen, J. Rimola, S. Schmidt, J. Stoker, J. Yang

**Affiliations:** 1 Centre for Medical Imaging, University College London UCL, Charles Bell House, 43-45 Foley Street, London, W1W 7TS UK; 2 Department of Radiology, St James’s University Hospital, Leeds Teaching Hospitals Trust, Leeds, UK; 3 Department of Radiology, Sackler Faculty of Medicine, Tel Aviv University, Chaim Sheba Medical Center, Tel-Hashomer, Israel; 4 Department of Radiology, Hôpital Robert-Debré, Reims, France; 5 Department of Radiology, Inje University College of Medicine, Haeundae Paik Hospital, Busan, South Korea; 6 Department of Radiological Sciences, Sapienza University of Rome, Policlinico Umberto I Hospital, Rome, Italy; 7 Department of Radiology, Beaumont Hospital, Dublin, Ireland; 8 Division of Abdominal Imaging, Beth Israel Deaconess Medical Center, Harvard Medical School, Boston, MA USA; 9 Colorectal Centre of Excellence, University Hospital of Southern Denmark, Vejle, Denmark; 10 Radiology Department, Hospital Clinic de Barcelona, Barcelona, Spain; 11 Department of Radiology, University Hospital, CHUV, Lausanne, Switzerland; 12 Department of Radiology and Nuclear Medicine, Amsterdam UMC, Academic Medical Centre, University of Amsterdam, Amsterdam, The Netherlands; 13 Department of Radiology, Concord Hospital, Sydney, Australia

**Keywords:** Guideline, Practice guideline, Anal fistula, Anus diseases, Anal sphincter

## Abstract

**Objectives:**

To develop imaging guidelines for patients with fistula-in-ano and other causes of anal sepsis.

**Methods:**

An expert group of 13 members of the European Society of Gastrointestinal and Abdominal Radiology (ESGAR) used a modified Delphi process to vote on a series of consensus statements relating to the imaging of patients with potential anal sepsis. Participants first completed a questionnaire to gather practice information and to help frame the statements posed.

**Results:**

In the first round of voting, the expert group scored 51 statements of which 45 (88%) achieved immediate consensus. The remaining 6 statements were redrafted following input from the expert group and consensus achieved for all during a second round of voting, including an additional statement drafted. No statement was rejected due to a lack of consensus. After redrafting to improve clarity, 53 individual statements were presented.

**Conclusion:**

These expert consensus statements can be used to guide appropriate indication, acquisition, interpretation and reporting of medical imaging for patients with potential fistula-in-ano and other causes of anal sepsis.

**Key Points:**

• *Medical imaging, notably magnetic resonance imaging, is used widely for the diagnosis and monitoring of fistula-in-ano and other causes of anal and perianal sepsis.*

• *While the indexed medical literature is clear that diagnostic accuracy is potentially excellent, this depends on competent image acquisition and interpretation.*

• *In order to facilitate this, the European Society of Gastrointestinal and Abdominal Radiology (ESGAR) has produced expert consensus guidelines regarding the imaging of fistula-in-ano and related conditions.*

**Electronic supplementary material:**

The online version of this article (10.1007/s00330-020-06826-5) contains supplementary material, which is available to authorized users.

## Introduction

Following a 2018 survey of its membership, the Research Committee of the European Society of Gastrointestinal and Abdominal Radiology (ESGAR) identified imaging of fistula-in-ano as a priority for guideline development. Prioritisation is contingent on the disease having considerable clinical burden, having considerable impact on abdominal imaging services, existing uncertainty regarding best imaging practice, and the existence of sufficient indexed research from which to develop an evidence-based guideline. The target audience for this guideline are radiologists interpreting medical imaging for diagnosis of fistula-in-ano and other causes of anal sepsis.

## Methods

These guidelines were developed using a robust and transparent methodology via the ESGAR guideline development process [1]. A “monodisciplinary” approach was adopted since the guideline concerns radiological practice predominantly, with a focus on technical aspects of image acquisition and protocols, not requiring input from other societies. Ethical permission was not required by the authors’ universities for systematic review of available medical literature.

### Guideline group selection

The ESGAR research committee appointed the guideline Chair (SH), who then selected a Deputy (DT). The remaining guideline authors (“expert group”) were then selected by the Chair and his Deputy from the ESGAR membership following a call for expressions of interest. Selection was influenced by requirement for prior peer-reviewed indexed publication in the field, combined with wide geographical representation. Ultimately, there were 13 group members, including the Chair and Deputy (and comprising the authorship of this article).

### Guideline development

Guideline development utilised a modified Delphi approach based on the RAND/UCLA appropriateness criteria [2]. The Chair produced a document detailing potential items for subsequent consensus which were modified following discussion with his Deputy. The Chair then performed a systematic literature search in order to establish the evidence base around individual items. The US National Library of Medicine PubMed journal citation database was searched on 26 July 2018 without date restriction using the string described in Appendix 1, returning 1260 items. The review aimed to identify diagnostic test accuracy studies that compared imaging tests against each other and/or an independent reference standard, in the clinical context of anal sepsis and/or related conditions. Accordingly, there were 1121 exclusions as follows: topic not anal sepsis, etc., or where no imaging data was reported (1021 exclusions); narrative review, guideline, commentary, correspondence (70 exclusions); insufficient data reported, defined as 10 or fewer subjects (e.g. case reports), and/or no diagnostic accuracy data presented (29 exclusions); paper could not be retrieved (1 exclusion). This left 139 research papers (Appendix 2). These papers were then summarised in an evidence table that presented the citation, the research question, a brief summary of the findings, and the Oxford evidence level [3]. This table was used as the basis to refine items for subsequent consensus and identify new topics of concern. The Chair and Deputy then developed a questionnaire that addressed these items via four broad groupings: experience with pertinent imaging modalities and their clinical indications; magnetic resonance (MR) imaging; anal endosonography; study reporting. This questionnaire was completed independently by all 13 expert group members (including the Chair and Deputy).

Informed by responses, the Chair then reframed questions as individual consensus statements that were posed to the group for “first-round” voting via a PowerPoint presentation (Microsoft Corporation). Each statement appeared on an individual slide, with members asked to reflect their opinion of the statement via one of five responses: “Disagree strongly”; “Disagree somewhat”; “Undecided”; “Agree somewhat”; “Agree strongly”. A free text option allowed members to express their reasoning. The Chair collated responses and defined consensus for individual statements that achieved either “Agree somewhat” or “Agree strongly” from at least 11 (i.e. 85%) members. Statements failing consensus were reframed by the Chair accounting for free text responses, in an attempt to incorporate members’ concerns. These reframed statements were considered via a “second round”, in concert with the evidence table. Members’ free text thinking around the prior statements was also presented on the slides to help other members understand why the original statement had failed to achieve consensus (this procedure was used instead of a face-to-face meeting, due to the wide international distribution of the expert group).

## Results

The lifetime experience of the expert group for interpreting MR examinations for anal/perianal sepsis was 3 members, 100–499 examinations; 3 members, 500–999 examinations; 7 members, > 1000 examinations. The median number of MR examinations reported annually by individual members for anal/perianal sepsis was 100–199, with four members reporting more than this. In contrast, it transpired that only 4 members performed and interpreted anal endosonography but the Chair decided to proceed with voting on this, anticipating that the statements may still prove useful to the readership. Similarly, only two group members reported a lifetime interpretation of 100 or more contrast examinations for anal/perianal sepsis. Indeed, six members reported having no experience of contrast examinations.

In the first round, all group members considered 41 individual statements (excluding 10 statements relating to anal endosonography). Five (12%) of these failed to reach consensus and were redrafted, after which consensus was achieved for all. The 4 members performing anal endosonography considered 10 additional statements, one (10%) of which failed to reach consensus; the redrafted statement achieved full consensus. An additional statement relating to the definition of horseshoe extension was then posed to the group, and achieved consensus. After collating second-round responses, the Chair drafted the resulting guideline document, which comprised 53 individual statements (one original statement was split to facilitate interpretation). This was then reviewed and ultimately approved by the expert group. The consensus statements, with their associated evidence level, are shown in Table 1.

**Table 1:** Final list of consensus statements agreed by more than 80% of the committee members:

1. Clinical indications for imaging	
• It is recommended that anal imaging (usually MRI) may be used to investigate potential anal sepsis. Imaging can both diagnose and classify fistula-in-ano. Imaging may also be used to investigate acute ischioanal abscess (*II*)	
• It is recommended that anal imaging (usually MRI) may be used to investigate potentially complex pilonidal sinus and hidradenitis suppurativa, where the differential diagnosis includes cryptoglandular infection (*III*)	
• It is recommended that anal imaging (usually MRI) may be used to investigate anal pain in immunocompromised patients and anal necrotising fasciitis, since imaging reveals whether sepsis is present, and its extent (*III*)	
• It is recommended that anal imaging (usually MRI) may be used to monitor the effect of therapy on anal sepsis in Crohn’s disease (e.g. biological/cellular/surgical). Imaging can also be used before medical treatment is commenced, to identify any abscess that may preclude biological therapy (*II*)	
2. Imaging modalities	
• MRI is recommended as the most clinically useful imaging modality to investigate potential anal sepsis (*II*)	
• It is not recommended to use CT scanning as the primary investigation for fistula-in-ano where there is access to MRI and/or anal US. However, CT is sometimes still performed where MRI and/or anal US is unavailable, or in an emergency setting. In such circumstances, CT may provide useful information regarding the presence and extent of sepsis when interpreted by experienced observers. Where performed, it is recommended that CT employ intravenous contrast (*V*)	
• It is not recommended to use contrast fistulography to investigate fistula-in-ano where there is access to MRI and/or anal US. However, fistulography is sometimes still performed where MRI and/or anal US is unavailable. In such circumstances, fistulography may provide useful information regarding the extent of sepsis when interpreted by experienced observers who are aware of its limitations (*V*)	
3. Radiologist experience	
• Competent interpretation of MRI to investigate fistula-in-ano requires prior experience. On average, interpretation of at least 50 mentored examinations is recommended to achieve reasonable competence for independent reporting (*II*)	
4. Patient preparation for MRI	
• It is not recommended that patients fast prior to MRI (*V*)	
• It is not recommended that rectal emptying and/or distension is used. An anal lumen marker (e.g. a catheter) is also not recommended (*V*)	
• It is not recommended that an intravenous smooth muscle relaxant (e.g. hyoscine n butylbromide) is used (*V*)	
• It is recommended that patients are scanned supine	
• It is recommended that 0.1 mmol/kg gadolinium is sufficient, where administered (*V*)	
• It is recommended that no special modifications are necessary to image children (*V*)	
5. MRI acquisition technique	
• It is recommended that field strengths of 1.5 T or 3 T may be used, according to local availability and radiologist preference (*V*)	
• It is recommended that a surface coil is used to enhance image quality (*III*)	
• It is not recommended that endoanal receiver coils are used because they are unnecessary to achieve acceptable imaging quality (*III*)	
• It is recommended that a minimal acceptable MRI examination for fistula-in-ano should include axial, coronal and sagittal planes, with the axial and coronal planes aligned to the anal canal axis (*III*)	
• It is recommended that for at least one acquisition, the field of view is sufficient to capture extensions remote from the primary track, to include the supralevator and ischioanal compartments. This may be achieved by using at least one acquisition with “whole-pelvis” coverage (*II*)	
6. MRI sequences	
• MRI aims to identify sepsis and its precise relationship to the sphincter complex and adjacent structures. Multiple different sequences and orientations can be used to achieve this and will vary according to the personal preferences of the radiologist and the MRI platform(s) used (*V*)	
• The planes and sequences considered mandatory by a majority of committee members were axial, coronal and sagittal T2-weighted (with or without fat suppression) (*V*)	
• Many other sequences and orientations may be considered optional, and used to further facilitate interpretation. For example, these may include fat-suppressed T1-weighted sequences with gadolinium enhancement, short T-1 inversion recovery (STIR) sequences and diffusion-weighted sequences (*V*)	
7. Examination timing	
• It is recommended that MRI is acquired 4 weeks or more after therapeutic examination under anaesthetic (EUA). Interpretation of MR imaging following surgical intervention is challenging because of difficulties distinguishing between cavities created by surgical drainage and extensions that have not been treated (*V*)	
8. Interpretation and reporting	
• It is recommended that the radiological report include the clinical details (*V*)	
• It is recommended that the radiological report include the Parks classification for any fistula identified (*V*)	
• It is recommended that the radiological report include the St. James classification if used locally and believed useful, but not at the expense of the Parks classification (*V*)	
• It is recommended that the total number of fistulas is stated where multiple fistulas are present. Each individual fistula should then be described in turn as per these guidelines (*V*)	
• It is recommended that the exact radial location of any fistula is identified using the “clock face” nomenclature. Fistula location may also be described with reference to the appropriate anal quadrant, but not at the expense of an exact description (*V*)	
• It is recommended that the exact radial location of the internal opening is identified using the “clock face” nomenclature. Location can also be described with reference to the appropriate anal quadrant, but not at the expense of an exact description (*V*)	
• It is recommended that the exact radial location of the external opening is identified using the “clock face” nomenclature. Location can also be described with reference to the appropriate anal quadrant, but not at the expense of an exact description (*V*)	
• It is recommended that the height of the internal opening should be described relative to adjacent structures (e.g. presumed level of dentate line, puborectalis, anal verge). An exact measurement (in mm) may also be provided if possible, but not at the expense of a relative description (*V*)	
• Occasionally a single primary fistula track may have multiple internal openings. Where this is the case, it is recommended that this fact is stated along with the number and location of internal openings. The same applies to multiple external openings (*V*)	
• Occasionally multiple fistula tracks may share a single internal opening. Where this is the case, it is recommended that this fact is stated along with the location of the internal opening. The same applies to shared external openings (*V*)	
• The length of the fistula may be reported if this can be measured with reasonable precision, but is optional and not mandatory (*V*)	
• It is recommended that whether a fistula contains a seton is reported (noting that setons are occasionally difficult to identify with certainty) (*V*)	
• It is recommended that the anatomical location of any extension(s) identified is reported, irrespective of whether it is inter-sphincteric, ischio-anal, supralevator, or in any other anatomical location (*V*)	
• In addition to reporting the anatomical location of any extension(s) identified, it is recommended that an indication of the size should be given. Since the morphology of extensions is variable and true volume measurement is difficult, maximal cavity diameter is often used to reflect size (*V*)	
• It is recommended that a “horseshoe” extension is defined radiologically as a semilunar region of sepsis that spreads in the horizontal plane either side of an internal opening, to involve two or more adjacent quadrants. Horseshoe extensions may be ischioanal, intersphincteric, or supralevator (V)	
• It is not recommended to describe individually all of those anatomical compartments that are free of disease. However, a general summary covering regions free of disease can be included if wished (*V*)	
• It is recommended to report less common findings when encountered. Such findings might include proctitis or sacral osteomyelitis, for example (*V*)	
• It is recommended that comment is made regarding the anatomical integrity of the external and internal sphincters, especially where there is evidence of sphincter disruption (perhaps contingent on prior fistula surgery) (*V*)	
• It is recommended that the report reference any prior MR imaging where this is available, and that it describes how the present examination relates to these (*V*)	
9. Recent developments	
• Fistula activity may be reflected by various imaging biomarkers, including MR T2 signal intensity and/or enhancement following gadolinium contrast. Assessment of activity is relatively novel and criteria for “active” vs. “inactive” disease need precise definition. Accordingly, while it is not mandatory to report fistula “activity”, it is recommended that this information is provided where deemed useful by the radiologist and/or referring clinicians (*III*)	
10. Anal endosonography	
• Competent interpretation of anal endosonography to investigate fistula-in-ano requires prior experience. On average, interpretation of at least 50 mentored examinations is recommended to achieve reasonable competence for independent performance and reporting (*V*)	
• It is recommended that patients require no special preparation and are usually examined in the left-lateral position. The prone position is an alternative (*III*)	
• Instillation of hydrogen peroxide into the fistula is not recommended	
• Generally, most patients tolerate the examination well and sedation/analgesia is not recommended (*V*)	
• It is recommended that a 360° axial endoprobe between 7 and 10 MHz is used. A convex endoanal axial probe (e.g. for prostatic imaging) can be used where a full 360° probe is unavailable (*V*)	
• 3D image acquisition is not recommended as it is unnecessary to achieve maximal diagnostic accuracy. However, it can be helpful where it is necessary to review the examination subsequently (*V*)	
• It is recommended that simple grey-scale imaging is sufficient for optimal diagnostic accuracy (*V*)	
• Children are a special case and it is recommended that practitioners be especially sensitive regarding the intimate and potentially distressing nature of the examination. It is recommended that practitioners provide as reassuring an environment as possible. In young children, who may not be able to cooperate, general anaesthesia may be considered to facilitate the examination (*V*)	
• In the context of fistula-in-ano, anal endosonography is usually deployed to answer specific clinical questions. It is recommended that MRI is the primary modality with which to image fistula-in-ano (*II*)	
• Given this, it is recommended that anal US is used where MRI is contraindicated, to image the internal opening with greater spatial resolution than MRI, and to assess prior or anticipated sphincter division (*V*)	

Evidence strength (Oxford Centre for Evidence Based medicine; reference [3]) shown in parentheses: *V*, expert opinion; *IV*, case control studies; *III*, inconsistent reference standard; *II*, cohort study with consistent reference standard; *I*, systematic review of level II studies

## Discussion

As noted in the “Introduction”, the primary target audience for this guideline are radiologists interpreting medical imaging for diagnosis of fistula-in-ano and other causes of anal sepsis. The group anticipate that these guidelines can be implemented rapidly in clinical practice since they predominantly describe MRI techniques and interpretation practice that are already in widespread clinical use. In particular, there is no specified requirement for specialised equipment. Rather, this guideline describes a “minimum dataset” for image acquisition and reporting.

Using a modified Delphi approach, we achieved consensus for 53 individual statements relating to imaging of potential anal sepsis, notably fistula-in-ano. These guidelines cover 10 areas of interest. The first concerns clinical indications. While this exercise was precipitated by a desire for guidelines around imaging fistula-in-ano, it is clear that imaging, notably MRI, is appropriate to investigate several other pathologies that may also cause anal sepsis. In some cases, differential diagnosis between fistula-in-ano and another pathology (e.g. pilonidal sinus) may be required, whereas in others imaging is highly effective for diagnosis of life-threatening pathology, notably necrotising fasciitis (where free tissue gas is especially well demonstrated by CT). In Crohn’s disease, imaging can also be used to direct and optimise treatment strategy and to monitor the efficacy of this. Several research studies have been performed that investigate the effect of imaging in the context of potential anal sepsis, and evidence of benefit reached level II standard (i.e. studies of consecutive patients compared with a robust reference standard).

Concerning the modalities employed, there was a clear preference for MRI. Several highly cited outcome studies of consecutive patients imaged using MRI have been published, confirming considerable diagnostic and therapeutic impact, and providing level II evidence of benefit [4–6]. The group encountered more difficulty when considering CT and contrast fistulography, which are now performed rarely by a large majority of members. However, we did conclude that these modalities have a role where MRI and/or anal endosonography is unavailable. In particular, group members indicated that CT was sometimes performed for suspected acute anal/perianal sepsis in emergency situations where MRI was unavailable (e.g. overnight). We make no statement regarding the level of experience required for competent interpretation of these modalities since personal and research evidence was lacking, in contrast to MRI [7].

Statements relating to patient preparation for anal MRI were largely based on expert opinion since little research has investigated this issue directly. In contrast, level III studies have investigated MRI acquisition techniques. One of the questions failing consensus initially was related to field strength. The first-round statement indicated a preference for 3.0-T platforms but several group members felt that these convey no additional diagnostic advantages over 1.5 T. Ultimately, we agreed both 1.5-T and 3.0-T platforms are sufficient for accurate diagnosis and choice will depend on local availability and personal preference; many members indicated no preference for either platform. The group agreed that axial and coronal acquisitions should always be aligned with respect to the anal axis since conventional acquisitions aligned with the table top result in oblique anal images that frustrate accurate diagnosis. These should always be combined with at least one acquisition whose field-of-view is sufficient to capture distant extensions (for example, supra-levator, pre-sacral disease). The questionnaire administered prior to the first round of voting made it abundantly clear that a plethora of different sequences were used across the group for daily clinical practice. Nevertheless, the majority of group members adopted T2-weighted sequences (with or without fat suppression) in axial, coronal and sagittal orientations. We were able to reach consensus by adopting a statement that recommended any sequence able to “identify sepsis and its precise relationship to the sphincter complex and adjacent structures”.

One issue familiar to practitioners is the difficulty encountered when MRI is performed soon after therapeutic examination under anaesthetic (EUA), when it can be problematic differentiating between treated and untreated disease. We were unable to identify any indexed research study that addressed this issue directly, so the recommendation that post-EUA MRI be delayed until at least 4 weeks was based on expert opinion alone.

The group was also able to reach consensus regarding a “minimum dataset” of information that should be contained within the imaging report, although these were based exclusively on expert opinion since we identified no research that investigated these items directly. However, a research article published by a group member subsequent to our literature search found that structured imaging reports for patients with fistula-in-ano missed fewer key features than narrative reports, and had greater utility for the referring clinician [8]. A further article from another group member advised a minimum dataset of items necessary for an informative report [9]. Accordingly, Table 2 describes those items that the present group considered comprise a minimum dataset for the reporting of MRI for fistula-in-ano. The aim is to present imaging information in a format useful to the referring clinician. For this reason, the group emphasised the importance of Parks’ classification [10] and clock-face nomenclature since these are concepts instantly familiar to clinicians and radiologists working in this field. The group decided that the exact radial location of any fistula should be reported, preferring this to the appropriate quadrant (although the latter can be included if wished). It should be noted that fistula tracks often follow a very tortuous route so that the internal and external openings may not be in the same quadrant, and the reporting radiologist may have to settle on an approximation for the location of the primary track overall. The fact that the group recommend the radial location of both internal and external openings be reported should account for this.

**Table 2:** Minimum dataset for the MRI reporting of fistula-in-ano

Item	
1. Clinical details	State the clinical question being asked of the radiologist.
2. Fistula	State whether a fistula is present or not (or sinus, or isolated abscess, etc.); its radial location (clock-face); its Parks classification; radial location and level of internal opening; radial location of external opening. Repeat for multiple fistulas. Describe shared internal/external openings, etc. State if seton present. Describe activity if used locally.
3. Extensions	State whether an extension(s) is present or not and its anatomical location. Indicate the maximal cavity diameter.
4. Sphincter integrity	Comment on internal and external sphincter integrity.
5. Associated findings	e.g., Proctitis, osteomyelitis
6. Comparison with prior imaging	Describe how imaging relates to prior studies where available.

The internal opening of most fistulas is at the dentate level, which is where the anal glands tend to congregate, emptying into the anal crypts, and these glands are believed to be the source of cryptoglandular sepsis [10]. The dentate line is a critical landmark and the radiological report should indicate the level of the internal opening relative to this (especially if higher). However, the problem is that the dentate line marks a histological boundary invisible on conventional imaging (the transition of modified columnar epithelium to squamous). Furthermore, anal canal length varies from person-to-person and is generally shorter in women. In general, the dentate line can be found approximately one-third along the anal canal length, cranial to the external anal verge. Inexperienced observers frequently estimate dentate level as higher than the real level. While acknowledging that no method is precise, for guidance, we suggest it is reasonable to estimate dentate line level relative to other structures: Methods might include measuring a distance between 1 to 2 cm cranial to the anal verge or by identifying the anal canal level a little caudal to the lowermost fibres of the puborectalis muscle. Some members provide the distance between the internal opening and the most caudal extent of the subcutaneous external sphincter (by measurement on coronal acquisitions).

The group encountered some difficulty when it came to indicating the size of any extension encountered since their morphology is variable. While volume measurements may circumvent this, volume is used rarely in daily clinical practice and is less familiar to surgeons. Ultimately, we settled on maximal diameter as the best compromise. This issue is similar to the distinction between an “abscess” and a “track / tract” because there is no generally accepted definition that differentiates between the two. Ultimately, accurate identification and location of sepsis is more important than the precise term used to describe it. There was also some discussion as to whether the maximal diameter of an extension should be restricted to its cavity or include any fibrous wall. Members decided that cavity measurement best reflected the need for treatment, e.g. surgical drainage.

First-round consensus failed for a statement regarding fistula “activity”. A number of recent publications have considered activity, notably in patients with Crohn’s fistulas [11–13]. The concept of activity is well established for enteric Crohn’s disease and when applied to fistula-in-ano is defined as inflammatory activity within the fistula reflected, for example, by T2 hyperintensity and/or enhancement following gadolinium contrast. While authors of these articles considered the topic important, other group members reported that their surgeons/gastroenterologists did not find this concept useful, being more concerned with whether a fistula was present or not, and its morphology. Ultimately, the group achieved consensus by recommending that activity be reported where local clinicians find the information helpful.

As noted earlier, it transpired that only four of the expert group performed anal endosonography but we nevertheless pressed ahead with consensus statements on this, believing some information to be preferable to no information at all. Ultimately, consensus was achieved for 10 statements, most of which were based on expert opinion alone, and which concluded that, overall, MRI is the preferred modality with which to image fistula-in-ano. Endosonography will have a specific role to image the internal opening with greater spatial resolution than MRI, and to assess prior or anticipated sphincter division. It is interesting to note that group opinion diverged from the indexed literature in places. This was most notable for hydrogen peroxide instillation: We identified 19 indexed articles describing hydrogen peroxide. Nine of these compared enhanced and unenhanced endosonography, with 7 finding enhancement beneficial. However, none of the group considered this adjunct necessary for accurate diagnosis. Similarly, none of the group used perineal US, presumably, because they have access to endosonography.

Concerning future research, since pre-operative classification of fistula-in-ano and similar pathology by imaging is already excellent, further gains in diagnostic accuracy are likely to be limited. Our consensus suggests that future research is likely to centre on the clinical utility of “activity” in the context of Crohn’s disease. These guidelines will be updated by ESGAR to account for these and other related developments.

## Electronic supplementary material

ESM 1 (DOCX 40 kb)

ESM 2 (DOCX 40 kb)

## Abbreviations

CT: Computed tomography
ESGAR: European Society of Gastrointestinal and Abdominal Radiology
EUA: Examination under anaesthetic
MRI: Magnetic resonance imaging
RAND/UCLA: Research and Development/University of California, Los Angeles
