# Supplementary material for: ESGAR consensus statement on the imaging of fistula-in-ano and other causes of anal sepsis
Source: Eur Radiol. 2020 Apr 19;30(9):4734–40. doi: 10.1007/s00330-020-06826-5 (PMC7431441; doi:10.1007/s00330-020-06826-5)
Supplement: Supplementary file 1 — (DOCX 40 kb) [file 330_2020_6826_MOESM1_ESM.docx]

Appendix 1.

The string used to search the US National Library of Medicine PUBMED journal citation database, limited to English language, and humans. Search performed 26^th^ July 2018.

(((((((((((((((((("magnetic resonance spectroscopy"[MeSH Terms] OR "magnetic resonance imaging"[MeSH Terms]) OR "magnetic resonance imaging"[MeSH Terms]) OR "magnetic resonance imaging"[MeSH Terms]) OR "tomography, x-ray computed"[MeSH Terms]) OR "tomography, spiral computed"[MeSH Terms]) OR "tomography, x-ray computed"[MeSH Terms]) OR helical ct[Other Term]) OR CT scan[Other Term]) OR volumetric CT[Other Term]) OR ct x ray[Other Term]) OR ("ultrasonography"[MeSH Terms] OR "ultrasonics"[MeSH Terms])) OR ultrasonography[Other Term]) OR ("ultrasonography"[MeSH Terms] OR "ultrasonography"[All Fields] OR ("ultrasound"[All Fields] AND "imaging"[All Fields]) OR "ultrasound imaging"[All Fields])) OR ("ultrasonography"[MeSH Terms] OR "ultrasonography"[All Fields] OR ("diagnostic"[All Fields] AND "ultrasound"[All Fields]) OR "diagnostic ultrasound"[All Fields])) OR (("ultrasonography"[MeSH Terms] OR "ultrasonography"[All Fields] OR "sonography"[All Fields]) AND medical[All Fields])) OR ("endosonography"[MeSH Terms] OR "endosonography"[All Fields])) OR ("endosonography"[MeSH Terms] OR "endosonography"[All Fields] OR ("endoscopic"[All Fields] AND "ultrasonography"[All Fields]) OR "endoscopic ultrasonography"[All Fields])) OR ("endosonography"[MeSH Terms] OR "endosonography"[All Fields] OR ("ultrasonic"[All Fields] AND "endoscopy"[All Fields]) OR "ultrasonic endoscopy"[All Fields])) OR ("endosonography"[MeSH Terms] OR "endosonography"[All Fields] OR ("echo"[All Fields] AND "endoscopy"[All Fields]) OR "echo endoscopy"[All Fields]) AND ((("rectal fistula"[MeSH Terms] OR fistula[Other Term]) OR "rectal fistula"[MeSH Terms]) OR fistula in ano[Other Term]) OR rectovaginal fistula[Other Term]
