# Supplementary material for: ESGAR consensus statement on the imaging of fistula-in-ano and other causes of anal sepsis
Source: Eur Radiol. 2020 Apr 19;30(9):4734–40. doi: 10.1007/s00330-020-06826-5 (PMC7431441; doi:10.1007/s00330-020-06826-5)
Supplement: Supplementary file 2 — (DOCX 40 kb) [file 330_2020_6826_MOESM2_ESM.docx]

**Appendix 2:**

**Indexed articles identified by literature search performed 26th July 2018.**

1. Luglio G, Giglio MC, Rispo A, Bucci C, Sollazzo V, Castiglione F, De Palma GD, Bucci L. Diagnostic Accuracy of 3-Dimensional Endoanal Ultrasound in Identifying Perianal Crohn's Fistulas. Dis Colon Rectum. 2018 Aug;61(8):931-937.
2. Lam D, Yong E, D'Souza B, Woods R. Three-Dimensional Modeling for Crohn's Fistula-in-Ano: A Novel, Interactive Approach. Dis Colon Rectum. 2018 May;61(5):567-572.
3. Garg P. Understanding and Treating Supralevator Fistula-in-Ano: MRI Analysis of 51 Cases and a Review of Literature. Dis Colon Rectum. 2018 May;61(5):612-621.
4. Lefrançois P, Zummo-Soucy M, Olivié D, Billiard JS, Gilbert G, Garel J, Visée E, Manchec P, Tang A. Diagnostic performance of intravoxel incoherent motion diffusion-weighted imaging and dynamic contrast-enhanced MRI for assessment of anal fistula activity. PLoS One. 2018 Jan 25;13(1):e0191822.
5. Emile SH, Magdy A, Youssef M, Thabet W, Abdelnaby M, Omar W, Khafagy W. Utility of Endoanal Ultrasonography in Assessment of Primary and Recurrent Anal Fistulas and for Detection of Associated Anal Sphincter Defects. J Gastrointest Surg. 2017 Nov;21(11):1879-1887.
6. Thomassin L, Armengol-Debeir L, Charpentier C, Bridoux V, Koning E, Savoye G, Savoye-Collet C. Magnetic resonance imaging may predict deep remission in patients with perianal fistulizing Crohn's disease. World J Gastroenterol. 2017 Jun 21;23(23):4285-4292.
7. Haliloglu N, Gulpinar B, Ozkavukcu E, Erden A. Typical MR imaging findings of perianal infections in patients with hematologic malignancies. Eur J Radiol. 2017 Aug;93:284-288.
8. Maconi G, Greco MT, Asthana AK. Transperineal Ultrasound for Perianal Fistulas and Abscesses - A Systematic Review and Meta-Analysis. Ultraschall Med. 2017 Jun;38(3):265-272.
9. Baik J, Kim SH, Lee Y, Yoon JH. Comparison of T2-weighted imaging, diffusion-weighted imaging and contrast-enhanced T1-weighted MR imaging for evaluating perianal fistulas. Clin Imaging. 2017 Jul - Aug;44:16-21.
10. Bezzio C, Bryant RV, Manes G, Maconi G, Saibeni S. New horizons in the imaging of perianal Crohn's disease: transperineal ultrasonography. Expert Rev Gastroenterol Hepatol. 2017 Jun;11(6):523-530.
11. Monnier L, Dohan A, Amara N, Zagdanski AM, Drame M, Soyer P, Hoeffel C. Anoperineal disease in Hidradenitis Suppurativa : MR imaging distinction from perianal Crohn's disease. Eur Radiol. 2017 Oct;27(10):4100-4109.
12. Ommer A, Herold A, Berg E, Fürst A, Post S, Ruppert R, Schiedeck T, Schwandner O, Strittmatter B. German S3 guidelines: anal abscess and fistula (second revised version). Langenbecks Arch Surg. 2017 Mar;402(2):191-201.
13. Garg P, Singh P, Kaur B. Magnetic Resonance Imaging (MRI): Operative Findings Correlation in 229 Fistula-in-Ano Patients. World J Surg. 2017 Jun;41(6):1618-1624.
14. Kołodziejczak M, Santoro GA, Obcowska A, Lorenc Z, Mańczak M, Sudoł-Szopińska I. Three-dimensional endoanal ultrasound is accurate and reproducible in determining type and height of anal fistulas. Colorectal Dis. 2017 Apr;19(4):378-384.
15. Oliveira IS, Kilcoyne A, Price MC, Harisinghani M. MRI features of perianal fistulas: is there a difference between Crohn's and non-Crohn's patients? Abdom Radiol (NY). 2017 Apr;42(4):1162-1168.
16. Bor R, Farkas K, Bálint A, Szűcs M, Ábrahám S, Milassin Á, Rutka M, Nagy F, Milassin P, Szepes Z, Molnár T. Prospective Comparison of Magnetic Resonance Imaging, Transrectal and Transperineal Sonography, and Surgical Findings in Complicated Perianal Crohn Disease. J Ultrasound Med. 2016 Nov;35(11):2367-2372.
17. Wortsman X, Castro A, Figueroa A. Color Doppler ultrasound assessment of morphology and types of fistulous tracts in hidradenitis suppurativa (HS). J Am Acad Dermatol. 2016 Oct;75(4):760-767.
18. Zhang H, Zhou ZY, Hu B, Liu DC, Peng H, Xie SK, Su D, Ren DL. Clinical Significance of 2 Deep Posterior Perianal Spaces to Complex Cryptoglandular Fistulas. Dis Colon Rectum. 2016 Aug;59(8):766-74.
19. Sirikurnpiboon S, Phadhana-anake O, Awapittaya B. Comparison of Endoanal Ultrasound with Clinical Diagnosis in Anal Fistula Assessment. J Med Assoc Thai. 2016 Feb;99 Suppl 2:S69-74.
20. Terracciano F, Scalisi G, Bossa F, Scimeca D, Biscaglia G, Mangiacotti M, Valvano MR, Perri F, Simeone A, Andriulli A. Transperineal ultrasonography: First level exam in IBD patients with perianal disease. Dig Liver Dis. 2016 Aug;48(8):874-9.
21. Pinson C, Dolores M, Cruypeninck Y, Koning E, Dacher JN, Savoye G, Savoye-Collet C. Magnetization transfer ratio for the assessment of perianal fistula activity in Crohn's disease. Eur Radiol. 2017 Jan;27(1):80-87.
22. Yin HQ, Wang C, Peng X, Xu F, Ren YJ, Chao YQ, Lu JG, Wang S, Xiao HS. Clinical value of endoluminal ultrasonography in the diagnosis of rectovaginal fistula. BMC Med Imaging. 2016 Apr 6;16:29.
23. Soker G, Gulek B, Yilmaz C, Kaya O, Arslan M, Dilek O, Gorur M, Kuscu F, İrkorucu O. The comparison of CT fistulography and MR imaging of perianal fistulae with surgical findings: a case-control study. Abdom Radiol (NY). 2016 Aug;41(8):1474-83.
24. Zhan S, Yang S, Lin J, Zhu Q, Lu F, Tan W, Cheng R, Gong Z, Yang W. Use of a Balloon Rectal Catheter in Magnetic Resonance Imaging of Complex Anal Fistula to Improve Detection of Internal Openings. J Comput Assist Tomogr. 2016 Jul-Aug;40(4):543-50.
25. Lo Re G, Tudisca C, Vernuccio F, Picone D, Cappello M, Agnello F, Galia M, Galfano MC, Biscaldi E, Salerno S, Pinto A, Midiri M, Lagalla R. MR imaging of perianal fistulas in Crohn's disease: sensitivity and specificity of STIR sequences. Radiol Med. 2016 Apr;121(4):243-51.
26. Visscher AP, Schuur D, Slooff RA, Meijerink WJ, Deen-Molenaar CB, Felt-Bersma RJ. Predictive factors for recurrence of cryptoglandular fistulae characterized by preoperative three-dimensional endoanal ultrasound. Colorectal Dis. 2016 May;18(5):503-9.
27. Ding JH, Bi LX, Zhao K, Feng YY, Zhu J, Zhang B, Yin SH, Zhao YJ. Impact of three-dimensional endoanal ultrasound on the outcome of anal fistula surgery: a prospective cohort study. Colorectal Dis. 2015 Dec;17(12):1104-12.
28. Brillantino A, Iacobellis F, Di Sarno G, D'Aniello F, Izzo D, Paladino F, De Palma M, Castriconi M, Grassi R, Di Martino N, Renzi A. Role of tridimensional endoanal ultrasound (3D-EAUS) in the preoperative assessment of perianal sepsis. Int J Colorectal Dis. 2015 Apr;30(4):535-42.
29. Waniczek D, Adamczyk T, Arendt J, Kluczewska E. Direct MRI fistulography with hydrogen peroxide in patients with recurrent perianal fistulas: a new proposal of extended diagnostics. Med Sci Monit. 2015 Feb 10;21:439-45.
30. de la Portilla F, Durán V, Maestre MV, Díaz-Pavón JM, Vázquez-Monchul JM, Palacios C, Gollonet JL, Sánchez-Gil JM. Effectiveness of a three-dimensional anorectal ultrasound in perianal Crohn's disease: incompatibility with clinical and surgical examinations. Int J Colorectal Dis. 2015 Apr;30(4):529-34.
31. Garcia-Granero A, Granero-Castro P, Frasson M, Flor-Lorente B, Carreño O, Espí A, Puchades I, Garcia-Granero E. Management of cryptoglandular supralevator abscesses in the magnetic resonance imaging era: a case series. Int J Colorectal Dis. 2014 Dec;29(12):1557-64.
32. Dohan A, Eveno C, Oprea R, Pautrat K, Placé V, Pocard M, Hoeffel C, Boudiaf M, Soyer P. Diffusion-weighted MR imaging for the diagnosis of abscess complicating fistula-in-ano: preliminary experience. Eur Radiol. 2014 Nov;24(11):2906-15.
33. Nagendranath C, Saravanan MN, Sridhar C, Varughese M. Peroxide-enhanced endoanal ultrasound in preoperative assessment of complex fistula-in-ano. Tech Coloproctol. 2014 May;18(5):433-8.
34. Shenoy-Bhangle A, Nimkin K, Goldner D, Bradley WF, Israel EJ, Gee MS. MRI predictors of treatment response for perianal fistulizing Crohn disease in children and young adults. Pediatr Radiol. 2014 Jan;44(1):23-9.
35. Liang C, Jiang W, Zhao B, Zhang Y, Du Y, Lu Y. CT imaging with fistulography for perianal fistula: does it really help the surgeon? Clin Imaging. 2013 Nov-Dec;37(6):1069-76.
36. Plaikner M, Loizides A, Peer S, Aigner F, Pecival D, Zbar A, Kremser C, Gruber H. Transperineal ultrasonography as a complementary diagnostic tool in identifying acute perianal sepsis. Tech Coloproctol. 2014 Feb;18(2):165-71.
37. Ziech ML, Lavini C, Bipat S, Ponsioen CY, Spijkerboer AM, Stokkers PC, Nederveen AJ, Stoker J. Dynamic contrast-enhanced MRI in determining disease activity in perianal fistulizing Crohn disease: a pilot study. AJR Am J Roentgenol. 2013 Feb;200(2):W170-7.
38. Kołodziejczak M, Sudoł-Szopińska I, Wilczyńska A, Bierca J. Utility of transperineal and anal ultrasonography in the diagnostics of hidradenitis suppurativa and its differentiation from a rectal fistula. Postepy Hig Med Dosw (Online). 2012 Nov 14;66:838-42.
39. Zbar AP, Horesh N, Bucholtz V, Zmora O, Beer-Gabel M, Carter D. Are there specific endosonographic features in Crohn's patients with perianal fistulae? J Crohns Colitis. 2013 Jul;7(6):490-6.
40. Siddiqui MR, Ashrafian H, Tozer P, Daulatzai N, Burling D, Hart A, Athanasiou T, Phillips RK. A diagnostic accuracy meta-analysis of endoanal ultrasound and MRI for perianal fistula assessment. Dis Colon Rectum. 2012 May;55(5):576-85.
41. Zawadzki A, Starck M, Bohe M, Thorlacius H. A unique 3D endoanal ultrasound feature of perianal Crohn's fistula: the 'Crohn ultrasound fistula sign'. Colorectal Dis. 2012 Sep;14(9):e608-11.
42. Garcés-Albir M, García-Botello SA, Esclapez-Valero P, Sanahuja-Santafé A, Raga-Vázquez J, Espi-Macías A, Ortega-Serrano J. Quantifying the extent of fistulotomy. How much sphincter can we safely divide? A three-dimensional endosonographic study. Int J Colorectal Dis. 2012 Aug;27(8):1109-16.
43. Lahat A, Assulin Y, Beer-Gabel M, Chowers Y. Endoscopic ultrasound for perianal Crohn's disease: disease and fistula characteristics, and impact on therapy. J Crohns Colitis. 2012 Apr;6(3):311-6.
44. Mullen R, Deveraj S, Suttie SA, Matthews AG, Yalamarthi S. MR imaging of fistula in ano: indications and contribution to surgical assessment. Acta Chir Belg. 2011 Nov-Dec;111(6):393-7.
45. Tozer P, Ng SC, Siddiqui MR, Plamondon S, Burling D, Gupta A, Swatton A, Tripoli S, Vaizey CJ, Kamm MA, Phillips R, Hart A. Long-term MRI-guided combined anti-TNF-α and thiopurine therapy for Crohn's perianal fistulas. Inflamm Bowel Dis. 2012 Oct;18(10):1825-34.
46. Horsthuis K, Ziech ML, Bipat S, Spijkerboer AM, de Bruine-Dobben AC, Hommes DW, Stoker J. Evaluation of an MRI-based score of disease activity in perianal fistulizing Crohn's disease. Clin Imaging. 2011 Sep-Oct;35(5):360-5.
47. Blom J, Nyström PO, Gunnarsson U, Strigård K. Endoanal ultrasonography may distinguish Crohn's anal fistulae from cryptoglandular fistulae in patients with Crohn's disease: a cross-sectional study. Tech Coloproctol. 2011 Sep;15(3):327-30.
48. Savoye-Collet C, Savoye G, Koning E, Dacher JN, Lerebours E. Fistulizing perianal Crohn's disease: contrast-enhanced magnetic resonance imaging assessment at 1 year on maintenance anti-TNF-alpha therapy. Inflamm Bowel Dis. 2011 Aug;17(8):1751-8.
49. Murad-Regadas SM, Regadas FS, Rodrigues LV, Fernandes GO, Buchen G, Kenmoti VT, Soares Gdos S, Holanda Ede C. Anatomic characteristics of anal fistula on three-dimensional anorectal ultrasonography. Dis Colon Rectum. 2011 Apr;54(4):460-6.
50. Yildirim N, Gökalp G, Öztürk E, Zorluoğlu A, Yilmazlar T, Ercan I, Savci G. Ideal combination of MRI sequences for perianal fistula classification and the evaluation of additional findings for readers with varying levels of experience. Diagn Interv Radiol. 2012 Jan-Feb;18(1):11-9.
51. Sudoł-Szopińska I, Kołodziejczak M, Szopiński TR. The accuracy of a postprocessing technique--volume render mode--in three-dimensional endoanal ultrasonography of anal abscesses and fistulas. Dis Colon Rectum. 2011 Feb;54(2):238-44.
52. Gligorijević V, Spasić N, Bojić D, Protić M, Svorcan P, Maksimovic B, Marković V, Krivokapić Z, Jojić Nj. The role of pelvic MRI in assesment of combined surgical and infliximab treatment for perianal Crohn's disease. Acta Chir Iugosl. 2010;57(3):89-95.
53. Karmiris K, Bielen D, Vanbeckevoort D, Vermeire S, Coremans G, Rutgeerts P, Van Assche G. Long-term monitoring of infliximab therapy for perianal fistulizing Crohn's disease by using magnetic resonance imaging. Clin Gastroenterol Hepatol. 2011 Feb;9(2):130-6.
54. Murad-Regadas SM, Regadas FS, Rodrigues LV, Holanda Ede C, Barreto RG, Oliveira L. The role of 3-dimensional anorectal ultrasonography in the assessment of anterior transsphincteric fistula. Dis Colon Rectum. 2010 Jul;53(7):1035-40.
55. Pomerri F, Dodi G, Pintacuda G, Amadio L, Muzzio PC. Anal endosonography and fistulography for fistula-in-ano. Radiol Med. 2010 Aug;115(5):771-83.
56. Subasinghe D, Samarasekera DN. Comparison of preoperative endoanal ultrasonography with intraoperative findings for fistula in ano. World J Surg. 2010 May;34(5):1123-7.
57. Hori M, Oto A, Orrin S, Suzuki K, Baron RL. Diffusion-weighted MRI: a new tool for the diagnosis of fistula in ano. J Magn Reson Imaging. 2009 Nov;30(5):1021-6.
58. Kim Y, Park YJ. Three-dimensional endoanal ultrasonographic assessment of an anal fistula with and without H(2)O(2) enhancement. World J Gastroenterol. 2009 Oct 14;15(38):4810-5.
59. Rosen MJ, Moulton DE, Koyama T, Morgan WM 3rd, Morrow SE, Herline AJ, Muldoon RL, Wise PE, Polk DB, Schwartz DA. Endoscopic ultrasound to guide the combined medical and surgical management of pediatric perianal Crohn's disease. Inflamm Bowel Dis. 2010 Mar;16(3):461-8.
60. Hutan M, Hutan M Jr, Satko M, Dimov A. Significance of MRI in the treatment of perianal fistula. Bratisl Lek Listy. 2009;110(3):162-5.
61. Losco A, Viganò C, Conte D, Cesana BM, Basilisco G. Assessing the activity of perianal Crohn's disease: comparison of clinical indices and computer-assisted anal ultrasound. Inflamm Bowel Dis. 2009 May;15(5):742-9.
62. Castellani D, Antonelli E, Sabatino G, Morelli O, Baldoni M, Clerici C, Morelli A, Bassotti G. A simplified method for anal ultrasonography: preliminary report. J Clin Gastroenterol. 2009 May-Jun;43(5):453-6.
63. Tepes B, Cerni I. The use of different diagnostic modalities in diagnosing fistula-in-ano. Hepatogastroenterology. 2008 May-Jun;55(84):912-5.
64. Toyonaga T, Tanaka Y, Song JF, Katori R, Sogawa N, Kanyama H, Hatakeyama T, Matsushima M, Suzuki S, Mibu R, Tanaka M. Comparison of accuracy of physical examination and endoanal ultrasonography for preoperative assessment in patients with acute and chronic anal fistula. Tech Coloproctol. 2008 Sep;12(3):217-23.
65. Weisman N, Abbas MA. Prognostic value of endoanal ultrasound for fistula-in-ano: a retrospective analysis. Dis Colon Rectum. 2008 Jul;51(7):1089-92.
66. Kleinübing H Jr, Jannini JF, Campos AC, Pinho M, Ferreira LC. The role of transperineal ultrasonography in the assessment of the internal opening of cryptogenic anal fistula. Tech Coloproctol. 2007 Dec;11(4):327-31.
67. Maconi G, Ardizzone S, Greco S, Radice E, Bezzio C, Bianchi Porro G. Transperineal ultrasound in the detection of perianal and rectovaginal fistulae in Crohn's disease. Am J Gastroenterol. 2007 Oct;102(10):2214-9.
68. Domkundwar SV, Shinagare AB. Role of transcutaneous perianal ultrasonography in evaluation of fistulas in ano. J Ultrasound Med. 2007 Jan;26(1):29-36.
69. Zbar AP, Oyetunji RO, Gill R. Transperineal versus hydrogen peroxide-enhanced endoanal ultrasonography in never operated and recurrent cryptogenic fistula-in-ano: a pilot study. Tech Coloproctol. 2006 Dec;10(4):297-302.
70. de la Portilla F, León-Jiménez E, Cisneros N, Rada R, Flikier B, Vega J, Hugo Maldonado V. Use of anorectal ultrasounds in perianal Crohn's disease: consistency with clinical data. Rev Esp Enferm Dig. 2006 Oct;98(10):747-54.
71. Fernández-Frías AM, Pérez-Vicente F, Arroyo A, Sánchez-Romero AM, Navarro JM, Serrano P, Oliver I, Costa D, Candela F, Calpena R. Is anal endosonography useful in the study of recurrent complex fistula-in-ano? Rev Esp Enferm Dig. 2006 Aug;98(8):573-81.
72. Caprioli F, Losco A, Viganò C, Conte D, Biondetti P, Forzenigo LV, Basilisco G. Computer-assisted evaluation of perianal fistula activity by means of anal ultrasound in patients with Crohn's disease. Am J Gastroenterol. 2006 Jul;101(7):1551-8.
73. Tilney HS, Heriot AG, Trickett JP, Massouh H, Edwards DP, Mellor SG, Gudgeon AM. The use of intra-operative endo-anal ultrasound in perianal disease. Colorectal Dis. 2006 May;8(4):338-41.
74. Toyonaga T, Matsushima M, Tanaka Y, Shimojima Y, Matsumura N, Kannyama H, Nozawa M, Hatakeyama T, Suzuki K, Yanagita K, Tanaka M. Microbiological analysis and endoanal ultrasonography for diagnosis of anal fistula in acute anorectal sepsis. Int J Colorectal Dis. 2007 Feb;22(2):209-13.
75. Mahjoubi B, Haizadch Kharazi H, Mirzaei R, Moghimi A, Changizi A. Diagnostic accuracy of body coil MRI in describing the characteristics of perianal fistulas. Colorectal Dis. 2006 Mar;8(3):202-7.
76. Maor Y, Chowers Y, Koller M, Zmora O, Bar-Meir S, Avidan B. Endosonographic evaluation of perianal fistulas and abscesses: comparison of two instruments and assessment of the role of hydrogen peroxide injection. J Clin Ultrasound. 2005 Jun;33(5):226-32.
77. Schwartz DA, White CM, Wise PE, Herline AJ. Use of endoscopic ultrasound to guide combination medical and surgical therapy for patients with Crohn's perianal fistulas. Inflamm Bowel Dis. 2005 Aug;11(8):727-32.
78. Ratto C, Grillo E, Parello A, Costamagna G, Doglietto GB. Endoanal ultrasound-guided surgery for anal fistula. Endoscopy. 2005 Aug;37(8):722-8.
79. Pascual Migueláñez I, García-Olmo D, Martínez-Puente MC, Pascual Montero JA. Is routine endoanal ultrasound useful in anal fistulas? Rev Esp Enferm Dig. 2005 May;97(5):323-7.
80. Szyszko TA, Bush J, Gishen P, Sellu D, Desouza NM. Endoanal magnetic resonance imaging of fistula-in-ano: a comparison of STIR with gadolinium-enhanced techniques. Acta Radiol. 2005 Feb;46(1):3-8.
81. Schaefer O, Lohrmann C, Kreisel W, Rasenack J, Ruf G, Hopt U, Langer M. Differentiation of perianal fistulas with digital subtraction magnetic resonance fistulography. Inflamm Bowel Dis. 2005 Apr;11(4):383-7.
82. Buchanan GN, Bartram CI, Williams AB, Halligan S, Cohen CR. Value of hydrogen peroxide enhancement of three-dimensional endoanal ultrasound in fistula-in-ano. Dis Colon Rectum. 2005 Jan;48(1):141-7.
83. West RL, Dwarkasing S, Felt-Bersma RJ, Schouten WR, Hop WC, Hussain SM, Kuipers EJ. Hydrogen peroxide-enhanced three-dimensional endoanal ultrasonography and endoanal magnetic resonance imaging in evaluating perianal fistulas: agreement and patient preference. Eur J Gastroenterol Hepatol. 2004 Nov;16(12):1319-24.
84. Al-Khawari HA, Gupta R, Sinan TS, Prakash B, Al-Amer A, Al-Bolushi S. Role of magnetic resonance imaging in the assessment of perianal fistulas. Med Princ Pract. 2005 Jan-Feb;14(1):46-52.
85. Buchanan GN, Halligan S, Bartram CI, Williams AB, Tarroni D, Cohen CR. Clinical examination, endosonography, and MR imaging in preoperative assessment of fistula in ano: comparison with outcome-based reference standard. Radiology. 2004 Dec;233(3):674-81.
86. Sudol-Szopinska I, Szczepkowski M, Panorska AK, Szopiński T, Jakubowski W. Comparison of contrast-enhanced with non-contrast endosonography in the diagnostics of anal fistulas. Eur Radiol. 2004 Dec;14(12):2236-41.
87. Buchanan GN, Halligan S, Taylor S, Williams A, Cohen R, Bartram C. MRI of fistula in ano: inter- and intraobserver agreement and effects of directed education. AJR Am J Roentgenol. 2004 Jul;183(1):135-40.
88. Ardizzone S, Maconi G, Colombo E, Manzionna G, Bollani S, Bianchi Porro G. Perianal fistulae following infliximab treatment: clinical and endosonographic outcome. Inflamm Bowel Dis. 2004 Mar;10(2):91-6.
89. Dwarkasing S, Hussain SM, Hop WC, Krestin GP. Anovaginal fistulas: evaluation with endoanal MR imaging. Radiology. 2004 Apr;231(1):123-8.
90. Navarro-Luna A, García-Domingo MI, Rius-Macías J, Marco-Molina C. Ultrasound study of anal fistulas with hydrogen peroxide enhancement. Dis Colon Rectum. 2004 Jan;47(1):108-14.
91. Rasul I, Wilson SR, MacRae H, Irwin S, Greenberg GR. Clinical and radiological responses after infliximab treatment for perianal fistulizing Crohn's disease. Am J Gastroenterol. 2004 Jan;99(1):82-8.
92. Moscowitz I, Baig MK, Nogueras JJ, Ovalioglu E, Weiss EG, Singh JJ, Wexner SD. Accuracy of hydrogen peroxide enhanced endoanal ultrasonography in assessment of the internal opening of an anal fistula complex. Tech Coloproctol. 2003 Oct;7(3):133-7.
93. Buchanan GN, Williams AB, Bartram CI, Halligan S, Nicholls RJ, Cohen CR. Potential clinical implications of direction of a trans-sphincteric anal fistula track. Br J Surg. 2003 Oct;90(10):1250-5.
94. Buchanan GN, Halligan S, Williams AB, Cohen CR, Tarroni D, Phillips RK, Bartram CI. Magnetic resonance imaging for primary fistula in ano. Br J Surg. 2003 Jul;90(7):877-81.
95. Sudol-Szopińska I, Jakubowski W, Szczepkowski M, Sarti D. Usefulness of hydrogen peroxide enhancement in diagnosis of anal and ano-vaginal fistulas. Eur Radiol. 2003 May;13(5):1080-4.
96. Chew SS, Yang JL, Newstead GL, Douglas PR. Anal fistula: Levovist-enhanced endoanal ultrasound: a pilot study. Dis Colon Rectum. 2003 Mar;46(3):377-84.
97. Taylor SA, Halligan S, Bartram CI. Pilonidal sinus disease: MR imaging distinction from fistula in ano. Radiology. 2003 Mar;226(3):662-7.
98. Van Assche G, Vanbeckevoort D, Bielen D, Coremans G, Aerden I, Noman M, D'Hoore A, Penninckx F, Marchal G, Cornillie F, Rutgeerts P. Magnetic resonance imaging of the effects of infliximab on perianal fistulizing Crohn's disease. Am J Gastroenterol. 2003 Feb;98(2):332-9.
99. Sudoł-Szopińska I, Geśla J, Jakubowski W, Noszczyk W, Szczepkowsi M, Sarti D. Reliability of endosonography in evaluation of anal fistulae and abscesses. Acta Radiol. 2002 Nov;43(6):599-602.
100. Buchanan G, Halligan S, Williams A, Cohen CR, Tarroni D, Phillips RK, Bartram CI. Effect of MRI on clinical outcome of recurrent fistula-in-ano. Lancet. 2002 Nov 23;360(9346):1661-2.
101. Maccioni F, Colaiacomo MC, Stasolla A, Manganaro L, Izzo L, Marini M. Value of MRI performed with phased-array coil in the diagnosis and pre-operative classification of perianal and anal fistulas. Radiol Med. 2002 Jul-Aug;104(1-2):58-67. English, Italian.
102. Sudoł-Szopińska I, Jakubowski W, Szczepkowski M. Contrast-enhanced endosonography for the diagnosis of anal and anovaginal fistulas. J Clin Ultrasound. 2002 Mar-Apr;30(3):145-50.
103. Stoker J, Rociu E, Schouten WR, Laméris JS. Anovaginal and rectovaginal fistulas: endoluminal sonography versus endoluminal MR imaging. AJR Am J Roentgenol. 2002 Mar;178(3):737-41.
104. Sloots CE, Felt-Bersma RJ, Poen AC, Cuesta MA, Meuwissen SG. Assessment and classification of fistula-in-ano in patients with Crohn's disease by hydrogen peroxide enhanced transanal ultrasound. Int J Colorectal Dis. 2001 Sep;16(5):292-7.
105. Schwartz DA, Wiersema MJ, Dudiak KM, Fletcher JG, Clain JE, Tremaine WJ, Zinsmeister AR, Norton ID, Boardman LA, Devine RM, Wolff BG, Young-Fadok TM, Diehl NN, Pemberton JH, Sandborn WJ. A comparison of endoscopic ultrasound, magnetic resonance imaging, and exam under anesthesia for evaluation of Crohn's perianal fistulas. Gastroenterology. 2001 Nov;121(5):1064-72.
106. Stewart LK, McGee J, Wilson SR. Transperineal and transvaginal sonography of perianal inflammatory disease. AJR Am J Roentgenol. 2001 Sep;177(3):627-32.
107. Beets-Tan RG, Beets GL, van der Hoop AG, Kessels AG, Vliegen RF, Baeten CG, van Engelshoven JM. Preoperative MR imaging of anal fistulas: Does it really help the surgeon? Radiology. 2001 Jan;218(1):75-84.
108. Sabir N, Sungurtekin U, Erdem E, Nessar M. Magnetic resonance imaging with rectal Gd-DTPA: new tool for the diagnosis of perianal fistula. Int J Colorectal Dis. 2000 Nov;15(5-6):317-22.
109. Ratto C, Gentile E, Merico M, Spinazzola C, Mangini G, Sofo L, Doglietto G. How can the assessment of fistula-inano be improved? Dis Colon Rectum. 2000 Oct;43(10):1375-82.
110. Chapple KS, Spencer JA, Windsor AC, Wilson D, Ward J, Ambrose NS. Prognostic value of magnetic resonance imaging in the management of fistula-in-ano. Dis Colon Rectum. 2000 Apr;43(4):511-6.
111. Madsen SM, Myschetzky PS, Heldmann U, Rasmussen OO, Thomsen HS. Fistula in ano: evaluation with low-field magnetic resonance imaging (0.1 T). Scand J Gastroenterol. 1999 Dec;34(12):1253-6.
112. Yee LF, Birnbaum EH, Read TE, Kodner IJ, Fleshman JW. Use of endoanal ultrasound in patients with rectovaginal fistulas. Dis Colon Rectum. 1999 Aug;42(8):1057-64.
113. Cho DY. Endosonographic criteria for an internal opening of fistula-in-ano. Dis Colon Rectum. 1999 Apr;42(4):515-8.
114. Orsoni P, Barthet M, Portier F, Panuel M, Desjeux A, Grimaud JC. Prospective comparison of endosonography, magnetic resonance imaging and surgical findings in anorectal fistula and abscess complicating Crohn's disease. Br J Surg. 1999 Mar;86(3):360-4.
115. Poen AC, Felt-Bersma RJ, Eijsbouts QA, Cuesta MA, Meuwissen SG. Hydrogen peroxide-enhanced transanal ultrasound in the assessment of fistula-in-ano. Dis Colon Rectum. 1998 Sep;41(9):1147-52.
116. Stoker J, Fa VE, Eijkemans MJ, Schouten WR, Laméris JS. Endoanal MRI of perianal fistulas: the optimal imaging planes. Eur Radiol. 1998;8(7):1212-6.
117. Halligan S, Bartram CI. MR imaging of fistula in ano: are endoanal coils the gold standard? AJR Am J Roentgenol. 1998 Aug;171(2):407-12.
118. Spencer JA, Chapple K, Wilson D, Ward J, Windsor AC, Ambrose NS. Outcome after surgery for perianal fistula: predictive value of MR imaging. AJR Am J Roentgenol. 1998 Aug;171(2):403-6.
119. deSouza NM, Gilderdale DJ, Coutts GA, Puni R, Steiner RE. MRI of fistula-in-ano: a comparison of endoanal coil with external phased array coil techniques. J Comput Assist Tomogr. 1998 May-Jun;22(3):357-63.
120. Halligan S, Healy JC, Bartram CI. Magnetic resonance imaging of fistula-in-ano: STIR or SPIR? Br J Radiol. 1998 Feb;71(842):141-5.
121. Poen AC, Felt-Bersma RJ, Cuesta MA, Meuwissen GM. Vaginal endosonography of the anal sphincter complex is important in the assessment of faecal incontinence and perianal sepsis. Br J Surg. 1998 Mar;85(3):359-63.
122. Mergo PJ, Helmberger T, Cerda JJ, Urrutia M, Ros PR. Rectal perflubron: new application in MRI of perirectal fistulae. J Comput Assist Tomogr. 1997 Mar-Apr;21(2):259-64.
123. Scholefield JH, Berry DP, Armitage NC, Wastie ML. Magnetic resonance imaging in the management of fistula in ano. Int J Colorectal Dis. 1997;12(5):276-9.
124. Spencer JA, Ward J, Beckingham IJ, Adams C, Ambrose NS. Dynamic contrast-enhanced MR imaging of perianal fistulas. AJR Am J Roentgenol. 1996 Sep;167(3):735-41.
125. Hussain SM, Stoker J, Schouten WR, Hop WC, Laméris JS. Fistula in ano: endoanal sonography versus endoanal MR imaging in classification. Radiology. 1996 Aug;200(2):475-81.
126. Haggett PJ, Moore NR, Shearman JD, Travis SP, Jewell DP, Mortensen NJ. Pelvic and perineal complications of Crohn's disease: assessment using magnetic resonance imaging. Gut. 1995 Mar;36(3):407-10.
127. Lunniss PJ, Barker PG, Sultan AH, Armstrong P, Reznek RH, Bartram CI, Cottam KS, Phillips RK. Magnetic resonance imaging of fistula-in-ano. Dis Colon Rectum. 1994 Jul;37(7):708-18.
128. Myhr GE, Myrvold HE, Nilsen G, Thoresen JE, Rinck PA. Perianal fistulas: use of MR imaging for diagnosis. Radiology. 1994 May;191(2):545-9.
129. Deen KI, Williams JG, Hutchinson R, Keighley MR, Kumar D. Fistulas in ano: endoanal ultrasonographic assessment assists decision making for surgery. Gut. 1994 Mar;35(3):391-4.
130. Barker PG, Lunniss PJ, Armstrong P, Reznek RH, Cottam K, Phillips RK. Magnetic resonance imaging of fistula-in-ano: technique, interpretation and accuracy. Clin Radiol. 1994 Jan;49(1):7-13.
131. Van Beers B, Grandin C, Kartheuser A, Hoang P, Mahieu P, Detry R, Vanheuverzwijn R, Pringot J. MRI of complicated anal fistulae: comparison with digital examination. J Comput Assist Tomogr. 1994 Jan-Feb;18(1):87-90.
132. Schratter-Sehn AU, Lochs H, Vogelsang H, Schurawitzki H, Herold C, Schratter M. Endoscopic ultrasonography versus computed tomography in the differential diagnosis of perianorectal complications in Crohn's disease. Endoscopy. 1993 Nov;25(9):582-6.
133. Cataldo PA, Senagore A, Luchtefeld MA. Intrarectal ultrasound in the evaluation of perirectal abscesses. Dis Colon Rectum. 1993 Jun;36(6):554-8.
134. Van Outryve MJ, Pelckmans PA, Michielsen PP, Van Maercke YM. Value of transrectal ultrasonography in Crohn's disease. Gastroenterology. 1991 Nov;101(5):1171-7.
135. Choen S, Burnett S, Bartram CI, Nicholls RJ. Comparison between anal endosonography and digital examination in the evaluation of anal fistulae. Br J Surg. 1991 Apr;78(4):445-7.
136. Tio TL, Mulder CJ, Wijers OB, Sars PR, Tytgat GN. Endosonography of peri-anal and peri-colorectal fistula and/or abscess in Crohn's disease. Gastrointest Endosc. 1990 Jul-Aug;36(4):331-6.
137. Kuhlman JE, Fishman EK. CT evaluation of enterovaginal and vesicovaginal fistulas. J Comput Assist Tomogr. 1990 May-Jun;14(3):390-4.
138. Law PJ, Talbot RW, Bartram CI, Northover JM. Anal endosonography in the evaluation of perianal sepsis and fistula in ano. Br J Surg. 1989 Jul;76(7):752-5.
139. Koelbel G, Schmiedl U, Majer MC, Weber P, Jenss H, Kueper K, Hess CF. Diagnosis of fistulae and sinus tracts in patients with Crohn disease: value of MR imaging. AJR Am J Roentgenol. 1989 May;152(5):999-1003.
